# A novel hybrid machine learning model for auxiliary diagnosing myocardial ischemia

**DOI:** 10.3389/fcvm.2024.1327912

**Published:** 2024-02-21

**Authors:** Jing Wang, Jing Xu, Jingsong Mao, Suzhong Fu, Haowei Gu, Naiming Wu, Guoqing Su, Zhiping Lin, Kaiyue Zhang, Yuetong Lin, Yang Zhao, Gang Liu, Hengyu Zhao, Qingliang Zhao

**Affiliations:** ^1^ Department of Imaging, School of Medicine, Xiamen Cardiovascular Hospital of Xiamen University, Xiamen University, Xiamen, China; ^2^ State Key Laboratory of Vaccines for Infectious Diseases, Center for Molecular Imaging and Translational Medicine, Xiang An Biomedicine Laboratory, Institute of Artificial Intelligence, School of Public Health, Innovation Laboratory for Sciences and Technologies of Energy Materials of Fujian Province (IKKEM), Xiamen University, Xiamen, China; ^3^ State Key Laboratory of Molecular Vaccinology and Molecular Diagnostics, National Innovation Platform for Industry-Education Integration in Vaccine Research, Xiamen University, Xiamen, China; ^4^ Department of Vascular Intervention, Affiliated Hospital, Guilin Medical University, Guilin, China; ^5^ Department of Radiology, Xiang’an Hospital of Xiamen University, Xiamen, China; ^6^ Department of Pharmaceutical Diagnosis, GE Healthcare, Guangzhou, China; ^7^ Department of Mechanical and Electrical Engineering, Xiamen University, Xiamen, China; ^8^ Shenzhen Research Institute of Xiamen University, Shenzhen, China

**Keywords:** coronary atherosclerosis, coronary CT angiography, radiomics, random forest model, medical image analysis

## Abstract

**Introduction:**

Accurate identification of the myocardial texture features of fat around the coronary artery on coronary computed tomography angiography (CCTA) images are crucial to improve clinical diagnostic efficiency of myocardial ischemia (MI). However, current coronary CT examination is difficult to recognize and segment the MI characteristics accurately during earlier period of inflammation.

**Materials and methods:**

We proposed a random forest model to automatically segment myocardium and extract peripheral fat features. This hybrid machine learning (HML) model is integrated by CCTA images and clinical data. A total of 1,316 radiomics features were extracted from CCTA images. To further obtain the features that contribute the most to the diagnostic model, dimensionality reduction was applied to filter features to three: LNS, GFE, and WLGM. Moreover, statistical hypothesis tests were applied to improve the ability of discriminating and screening clinical features between the ischemic and non-ischemic groups.

**Results:**

By comparing the accuracy, recall, specificity and AUC of the three models, it can be found that HML had the best performance, with the value of 0.848, 0.762, 0.704 and 0.729.

**Conclusion:**

In sum, this study demonstrates that ML-based radiomics model showed good predictive value in MI, and offer an enhanced tool for predicting prognosis with greater accuracy.

## 1 Introduction

The incidence and mortality rates of coronary heart disease have shown a continuous increasing trend, making it a leading cause of all-cause mortality (1). Whether coronary heart disease occurs when coronary arteries are atherosclerotic depends to some extent on the degree of narrowing of the blood vessels caused by atherosclerotic plaques and their stability (2–5). Coronary angiography (CAG) is an invasive imaging examination used to detect whether there is stenosis or occlusion in the coronary arteries. While it cannot provide detail such as arterial wall thickness and plaque characteristics, and it is weak in evaluating plaque characterization (6, 7). To address this limitation, intravascular ultrasound (IVUS) uses an ultrasound probe placed within the coronary arteries to provide high-resolution dynamic images, assisting doctors in assessing the structure of the arterial wall and distribution of plaques (8). However, it can only provide images of localized areas within the blood vessels and cannot comprehensively observe the entire coronary artery system.

**Figure 1:**
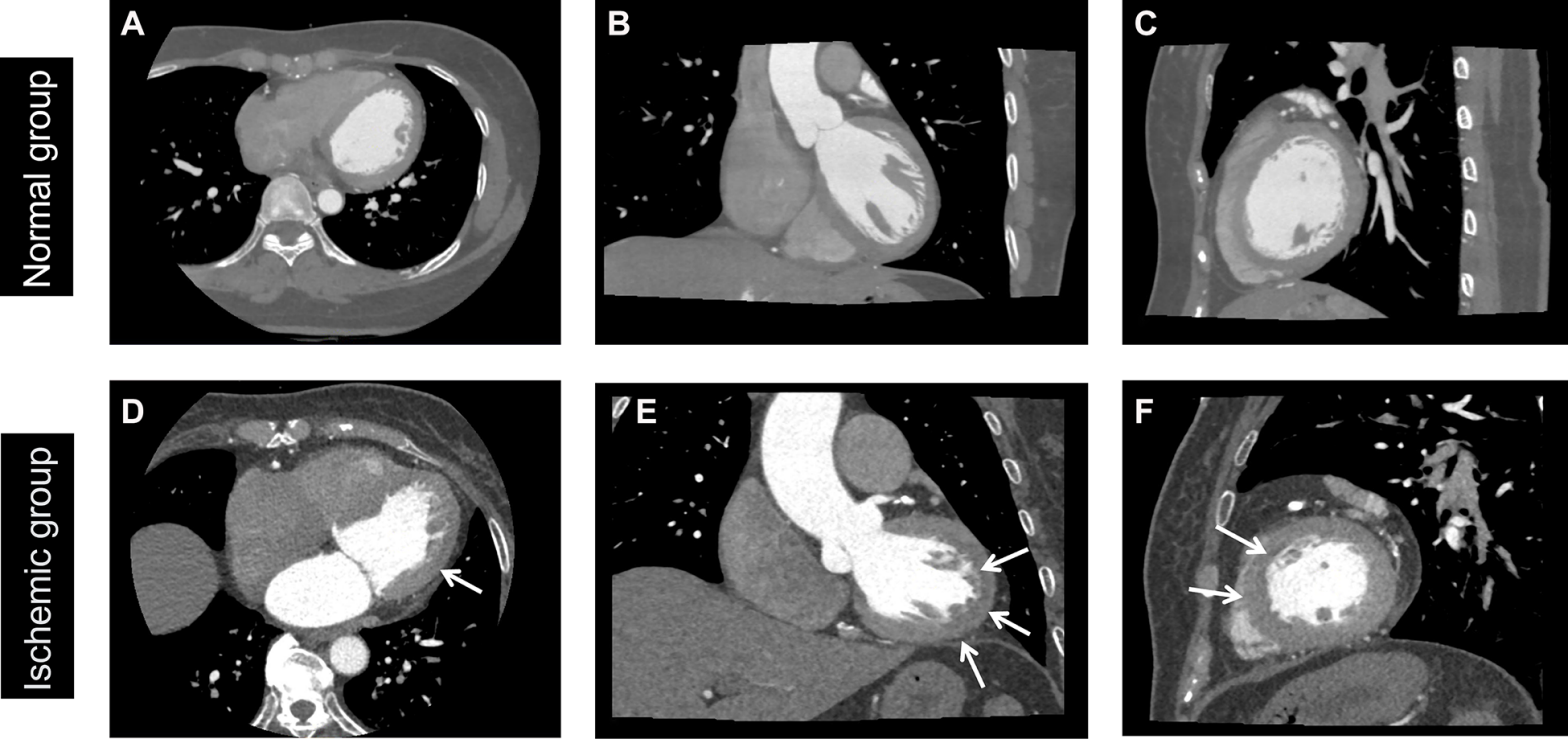
Normal and ischemic myocardium. Normal group: A 45-year-old female patient without MI. Ischemic group: a 65-year-old male patient with myocardial ischemia, as shown by the arrow. (**A,D**) Axial image, (**B,E**) coronal image, and (**C,F**) sagittal image. The window level was set to 100 HU, and the window width to 800 HU. Blue arrows are represented myocardial ischemic focus in **D**, **E** and **F**.

CCTA combines the advantages of both coronary angiography and computed tomography imaging in a mature, non-invasive imaging method (9). It makes regions of interest (ROI) more clearly from different angles, generating high-resolution 3D images to display the clear structure of the heart and coronary blood vessels (Figure 1). With its high sensitivity and negative predictive value, CCTA has been widely used in the diagnosis and treatment of coronary heart disease (10, 11). However, on the one hand, other effective methods need to be combined with CCTA to diagnose myocardial ischemia because of contrast agents or heart motion. On the other hand, the information in CCTA images depends on subjective judgment. Therefore, it is necessary to find a method that can quantitatively extract CCTA information to reduce the limitations of subjective judgment.

Based on the above, many studies have been devoted to solving this problem. From the perspective of traditional image processing, studies (12) have combined hessian filtering and local geometric features to track coronary arteries in CCTA using traditional image processing methods. There are also studies (13) utilizing radiomics, plaque segmentation is applied to compute diverse features related to shape, intensity, and texture under various image transformations. In recent years, artificial intelligence (AI) technology based on deep learning and computer vision has been widely used in various diagnosis and treatment services in the medical industry (14–16).

Inspired by the significant effects of the above methods on CCTA image processing, it is considered that combining radiomics-extracted tissue features with machine learning methods can construct a prediction model for automatic analysis of features such as narrow regions and calcification degree (17). At the same time, it can realize the rapid automation analysis of CCTA images, greatly improving the efficiency and accuracy of medical diagnosis.

## 2 Materials and methods

### 2.1 Data acquisition

We conducted a retrospective analysis of patient data from Xiamen Cardiovascular Hospital Affiliated to Xiamen University who underwent CCTA examination between January 2022 and December 2022. To ensure the suitability of clinical and imaging data for our research purposes, we established the following inclusion criteria: (1) the time interval between CCTA image acquisition and the diagnosis of myocardial ischemia is less than two weeks. (2) no myocardial infarction patients within the past three months or patients with typical or atypical angina symptoms for less than two months. (3) CCTA images do not have obvious motion artifacts or metal artifacts; and (4) no patients with other heart diseases. Exclusion criteria included: (1) patients with a history of coronary artery bypass grafting or stent implantation, (2) patients with a high heart rate (heart rate greater than 85 beats per minute or arrhythmia), and (3) patients with severe liver or kidney disease (18). In this study, the diagnosis of myocardial ischemia was based on clinical diagnosis and/or single photon emission computed tomography imaging diagnosis. A total of 158 samples were collected, with 70 patients diagnosed with ischemia and 88 age-matched healthy individuals serving as controls who underwent CCTA scans under equivalent conditions. We also collected clinical data from these individuals, including 8 characteristics: gender, age, history of hypertension, history of hyperlipidemia, history of diabetes, family history of heart disease, cardiac enzymes, and muscle calcium protein.

All patients were scanned using a 560-layer multi-spiral cardiovascular CT device (CardioGrapheTM; GE Healthcare) on an empty stomach. The scanning range was from the tracheal ridge to the bottom of the heart. The scan parameters were as follows: tube voltage 120 kVp, tube current 50 mA, CT rotation time 0.24 s, and a reconstruction layer thickness of 0.5 mm. An iodine contrast agent was injected intravenously using a high-pressure injector (Salient; Imaxeon Pty Ltd.), and the scan began 5 s after the trigger threshold was reached. The contrast agent used was omnipaque (350 mg I/ml, GE Healthcare), and the injection was performed according to the following protocol: coronary artery drug injection time of 12 s; for heart rates ≥75 beats/min, the rate increased by 0.2 ml/s for every 5 beats/min increase, and the dose increased accordingly. A physiological saline solution with a dose of 30 ml was injected at the same rate as the contrast agent. The CCTA scan was performed in accordance with the Society of Cardiovascular Computed Tomography (SCCT) Coronary Artery Computed Tomography Angiography Performance and Acquisition Guidelines in 2016, and the scan parameters followed the “As Low As Reasonably Achievable” (ALARA) principle.

### 2.2 CCTA images preprocessing

**Figure 2:**
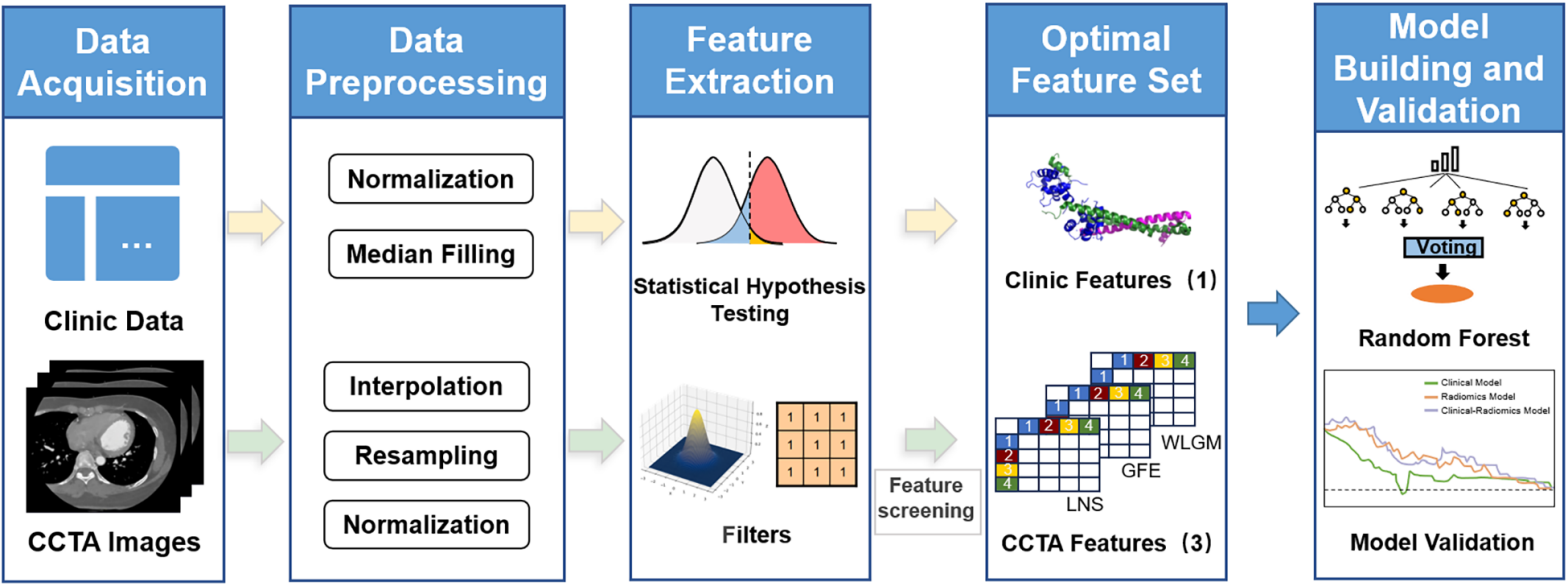
Flowchart of research based on radiomics and machine learning. Starting with clinical data and CCTA image data, statistical assumptions and radiomics are used to extract features from the two parts of data. After feature selection, a random forest model is established.

The study of radiomics can be divided into five steps: data preprocessing, image processing, feature extraction, exploratory analysis, and modeling (Figure 2). The purpose of data preprocessing is to improve the quality of the image, remove interference information such as noise and aliasing caused by human factors (19, 20). Specific methods include resampling, denoising, and data augmentation. First of all, medical images possess a large volume and necessitate adequate storage and processing capabilities. Furthermore, varying devices adhere to distinct image resolution standards, often resulting in non-uniform medical image resolutions. Therefore, it is necessary to use interpolation for resampling before processing the data. This method calculates new pixel values by interpolation calculation between pixel points, thus obtaining a feature and resolution-changed image. Common interpolation methods include linear interpolation, nearest neighbor interpolation, and so on. Furthermore, denoising is an essential step in the preprocessing process to enhance image quality and clarity. Depending on the corresponding CCTA image's quality, three methods can be employed for denoising: gaussian filter, median filter, and bilateral filter.

### 2.3 Features extraction and selection

Features can be extracted from the original image, including its shape features, gray-level co-occurrence matrix, gray-level run-length matrix, gray-level size zone matrix feature, and gray-level dependence matrix. In addition, these features can be re-extracted from the image for further analysis (21–24). Similarly, we can also perform wavelet transform, exponential transform, logarithmic transform, gradient transform, square transform, square root transform and other transforms on the original features except for the shape features. These transforms aim to modify the distribution of pixel values in an image and adjust its contrast, brightness, tone and other attributes. There are three main feature selection methods used in this study, namely low variance filtering, maximum relevance and minimum redundancy (mRMR) (25) and recursive feature elimination (RFE). We used variance-based dimensionality reduction for initial feature selection of radiomics features. Specifically, variance reduction reduces dimension by removing features with less variance. Variance represents the degree of dispersion of the feature, and if a feature has a small variance, it may contribute less to distinguishing the sample, so consider removing it. In addition, we used mRMR to reduce redundancy between features while preserving features highly associated with myocardial ischemia. Specifically, we calculate the mutual information between features to represent the degree of relevance, as follows:

$$I(x;y)=\int \;\;\;\int p(x,y)log\frac{\;p(x,y)}{\;p(x)p(y)}$$

Suppose that the feature set is $S\;=\;\{x_{1},x_{2},\ldots ,x_{n}\}$, in order to select the feature with the greatest correlation, S satisfies:

$$maxD(S,c),\;D=\frac{1}{\left|S\right|}\Sigma x_{i}\in S\;I(x_{i};c),$$

where *c* is the target feature myocardial ischemia. Secondly, to ensure the low redundancy of the set, the following formula needs to be satisfied:

$$\min R(S),\;R=\frac{1}{{\left|S\right|}^{2}}\Sigma x_{j},\;xi\in S\;I(x_{i};x_{j}),$$

The ultimate goal is to find the set S with the greatest degree of correlation and the least degree of redundancy, directly optimizing the following formula:

maxΦ(D,R),Φ=D−R.

Intuitively, an increase in D or R will both increase the objective function. The above process helped us reduce the number of features from 1,316 to 20. Too many features would increase the complexity of the model and the risk of overfitting. Therefore, we introduce RFE to reduce the final CCTA image radiomic features to 3. These methods aim to select the most informative features to generate the optimal feature set.

### 2.4 Statistical analysis

Unlike images, clinical data is usually stored in tabular form. The clinical data used in this study includes 8 characteristics: gender, age, history of hypertension, history of hyperlipidemia, history of diabetes, family history of heart disease, cardiac enzymes, and muscle calcium protein. To perform differential statistical analysis for each clinical feature, we use chi-square test, Mann–Whitney *U*-test, and *t*-test (for normal distribution). The chi-square test is utilized to examine the presence of a relationship between two discrete variables. For continuously distributed variables that conform to a normal distribution, *t*-tests are employed to compare the significance of differences in means between two independent samples. In cases where the features do not adhere to a normal distribution, we introduce the Mann–Whitney *U*-test, also known as the Wilcoxon rank-sum test. Generally, when the *p*-value is less than 0.05 or 0.01, it indicates a statistically significant difference between the two features; conversely, if the *p*-value exceeds the significance level, there is no evidence of a significant difference between the two features.

### 2.5 Classification model based on random forest

Through the above research process, two parts of features related to coronary heart disease can be extracted: CCTA image features and clinical features. A classification model named HML is constructed by combining these two parts of features. We consider using a random forest to establish a coronary heart disease classification model. A random forest is an ensemble learning method based on decision trees, whose basic idea is to build multiple decision trees and improve the performance of the model by voting or averaging (26, 27). In a random forest, a random subset of data samples and features is first selected. Then, this data and features are used to construct a set of decision trees.

Each decision tree consists of several layers of nodes, with each node serving as a judgment condition that determines which branch to which data should be directed within the tree. In the end, the majority voting mechanism or averaging method is employed to predict the category to which each sample belongs.

## 3 Results

### 3.1 CCTA features extraction

**Figure 3:**
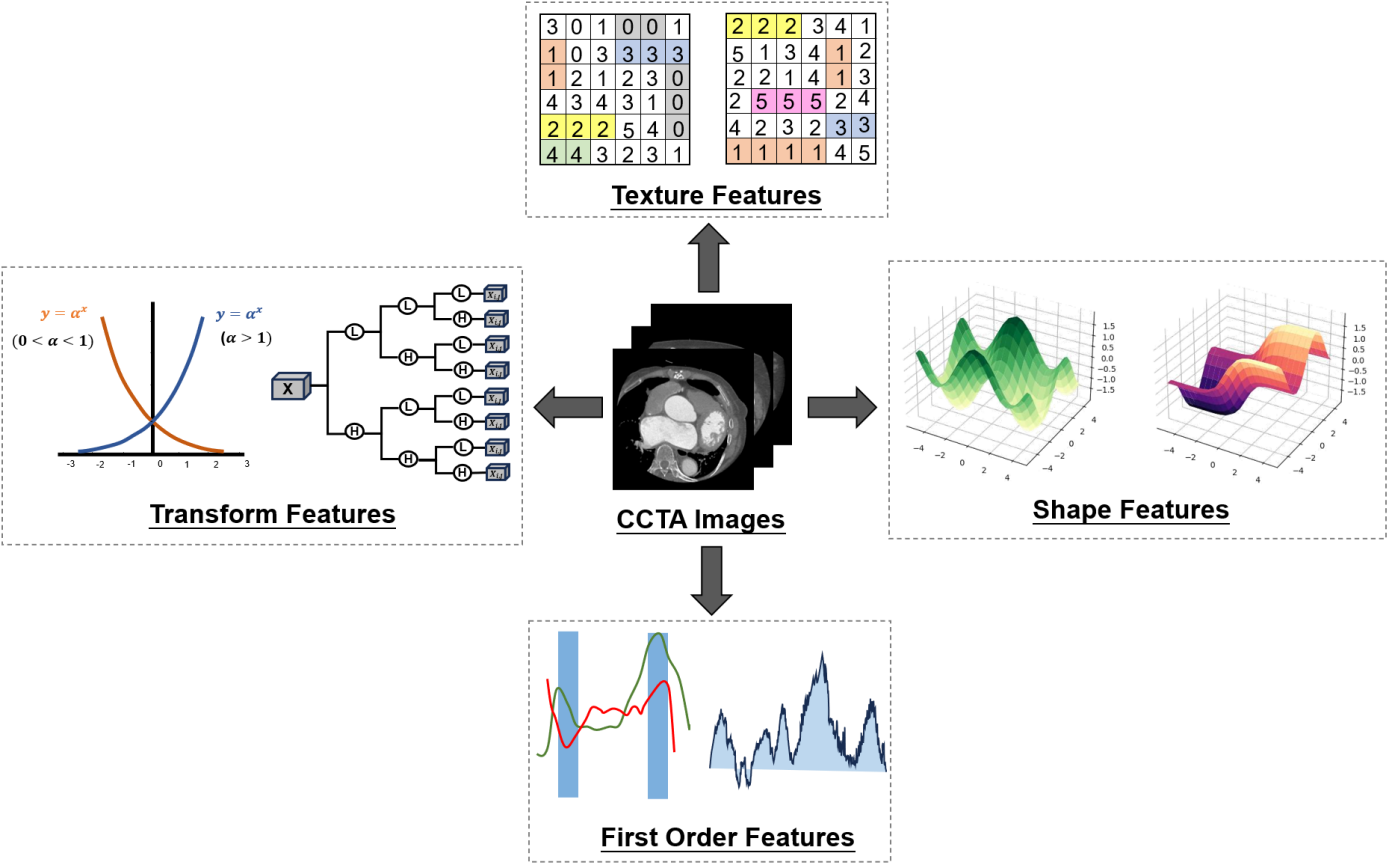
Radiomic features. Four kinds of characteristics of CCTA were extracted by radiomics. Then variance reduction and maximum correlation minimum redundancy reduction are used to determine the final optimal selection by recursive elimination.

Extensive radiomic feature extraction of CCTA can extract detailed features of the lesion area, including the size, shape, texture and other aspects of the disease description. Some features may be difficult to detect artificially. The universality of features provides the guarantee and foundation for the subsequent model building of machine learning. The screening process of 1,316 features of specific radiomics is as follows. The screening of radiomic features is based on Pyradiomics library in Python. Firstly, 107 original features of CCTA images are extracted. These include 15 shape features, 18 first order features, 24 gray level co-occurrence matrix (GLCM) features, 16 gray level runs length matrix (GLRLM) features, 18 gray level size zone matrix (GLSZM) features and 16 gray level dependence matrix (GLDM) features. Further, the Pyradiomics package provides us with a number of filters that can be used for re-extraction based on the original features. The first is the wavelet transform. In addition to shape features, the remaining features can be extracted again after the wavelet transform (107–14 = 93). The wavelet transform has 8 types of features LLH, LHL, LHH, HLL, HLH, HHL, HHH, LLL, so 93*8 = 744 features can be extracted after the wavelet transform. The same goes for Exponential, Gradient, Logarithm, Square, SquareRoot. After these five transform filters, 465 features can be obtained. Thus, a total of 1,316 radiomic features were obtained to provide medical features for subsequent machine learning models (107 + 93*8 + 93*5 = 1,316). The radiomics features obtained from CCTA images are classified into four categories: transform features, texture features, shape features and first order features (Figure 3).

### 3.2 Features selection

We used variance-based dimensionality reduction and mRMR method for initial feature selection of radiomics features, resulting in identification of the following 20 features (Table 1). While many features can enhance the model's learning capacity, they may also increase its complexity and lead to overfitting. To balance the above issues, we introduced recursive feature elimination to further select features, and obtained the final three CCTA image radiomics features: logarithm_ngtdm_Strength (LNS), wavelet-LLH_glcm_MCC (WLGM), and gradient_firstorder_Energy (GFE).

**Table 1:** Selected features by variance and REF.

Name of Features	
‘original_firstorder_Skewness’	‘wavelet-LLH_glcm_MCC'
’squareroot_glszm_GrayLevelVariance'	‘wavelet-HLL_firstorder_Skewness’
‘wavelet-HHL_firstorder_RobustMeanAbsoluteDeviation'	‘wavelet-LHL_glrlm_ShortRunHighGrayLevelEmphasis’
‘original_shape_Maximum2DDiameterColumn'	‘wavelet-LHH_firstorder_Entropy'
‘logarithm_ngtdm_Strength'	‘wavelet-LHL_glcm_Idn'
‘wavelet-HHH_glszm_SmallAreaLowGrayLevelEmphasis’	‘gradient_firstorder_Energy'
’squareroot_firstorder_Minimum'	‘wavelet-HHH_glszm_SizeZoneNonUniformity'
‘original_glszm_GrayLevelVariance'	‘wavelet-HLH_firstorder_Variance'
‘original_gldm_DependenceVariance'	‘wavelet-HHH_gldm_HighGrayLevelEmphasis’
‘wavelet-LLL_glcm_MaximumProbability'	‘wavelet-LLH_firstorder_Range'

The first feature represents the gradient strength calculated using a feature extraction method based on the laplacian operator after taking the logarithm of the original image. When there are distinct variations in gray intensity between brightness changes, indicating clear and well-defined features in the image, the value of strength tends to be high.

The second feature represents the calculation of the maximum correlation coefficient (MCC) using a feature extraction method based on gray-level co-occurrence matrix (GLCM) after applying low-low-high (LLH) wavelet transformation to the original image. When the texture feature is more complex, the value of MCC is usually larger, while when the texture feature is simple.

The third feature represents the calculation of energy in the first-order feature after applying gradient transformation to the original image. It is a measurement of the pixel value magnitude in the image. A slightly larger energy value may indicate the presence of some high-intensity pixels.

### 3.3 Statistical analysis of clinical data

To explore the statistical significance of clinical data on coronary heart disease, we conducted different statistical tests on each clinical feature in the normal and ischemic groups. For discrete variables, we used the chi-square test to compare the relationship between different clinical features and the presence of coronary heart disease. For continuous variables that meet normal distribution, we used the *t*-test to determine the correlation. For continuous variables that do not meet normal distribution, we used the Mann–Whitney *U*-test.

These three methods are non-parametric statistical tests based on certain assumptions to determine whether there are significant differences among multiple samples. In this study, a total of 88 patients with normal myocardial blood supply and 70 patients with myocardial ischemia were enrolled, of which 110 samples were used as the training set and 48 samples were used as the testing set. There were no statistical differences in all clinical features between the training and testing sets (*P*-value > 0.05), indicating that the clinical feature distributions of the two data sets were similar (Table 2). Only troponin showed a significant difference between the ischemic and healthy groups (*P*-value < 0.05) among all clinical features (Table 3), which can be explained medically. In coronary heart disease, myocardial ischemia and hypoxia caused by narrowing or blockage of coronary arteries can lead to myocardial cell damage and death, resulting in an increase in troponin concentration. Therefore, it is both reasonable to incorporate troponin concentration as a feature in the subsequent model.

**Table 2:** Statistical analysis of train group and test group.

Variable	Sample	Test	Train	Statistics	*P*-value
Female	58	21 (43.75%)	37 (33.64%)	1.471	0.225
Male	100	27 (56.25%)	73 (66.36%)		
Non_hypertension	53	17 (35.42%)	36 (32.73%)	0.108	0.742
Hypertension	105	31 (64.58%)	74 (67.27%)		
Non_hyperlipidemia	106	32 (66.67%)	74 (67.27%)	0.006	0.941
Hyperlipidemia	52	16 (33.33%)	36 (32.73%)		
Non_diabetes	99	29 (60.42%)	70 (63.64%)	0.148	0.7
Diabetes	59	19 (39.58%)	40 (36.36%)		
Non_family history of heart disease	97	32 (66.67%)	65 (59.09%)	0.809	0.368
Family history of heart disease	61	16 (33.33%)	45 (40.91%)		
Age	158	62.56 ± 11.33	62.13 ± 11.70	0.217	0.828
Cardiac enzymes	158	93.60 (66.38, 118.26)	101.30 (68.40, 118.26)	−0.391	0.696
Troponin	158	12.15 (6.85, 44.77)	15.20 (7.47, 51.69)	−0.556	0.578

**Table 3:** Statistical analysis of normal and ischemic.

Variable	Sample	Ischemic	Normal	Statistics	*P*-value
Female	58	38 (43.18%)	20 (28.57%)	3.582	0.058
Male	100	50 (56.82%)	50 (71.43%)		
Non_hypertension	53	33 (37.50%)	20 (28.57%)	1.394	0.238
Hypertension	105	55 (62.50%)	50 (71.43%)		
Non_hyperlipidemia	106	57 (64.77%)	49 (70.00%)	0.482	0.487
Hyperlipidemia	52	31 (35.23%)	21 (30.00%)		
Non_diabetes	99	57 (64.77%)	42 (60.00%)	0.38	0.538
Diabetes	59	31 (35.23%)	28 (40.00%)		
Non_family history of heart disease	97	51 (57.95%)	46 (65.71%)	0.99	0.32
Family history of heart disease	61	37 (42.05%)	24 (34.29%)		
Age	158	61.67 ± 11.26	63.00 ± 11.95	−0.718	0.474
Cardiac enzymes	158	93.60 (68.40, 118.26)	103.70 (68.24, 118.27)	−0.467	0.64
Troponin	158	10.60 (6.28, 29.62)	19.65 (8.58, 51.69)	−2.679	0.007

### 3.4 Random forest classification model

**Figure 4:**
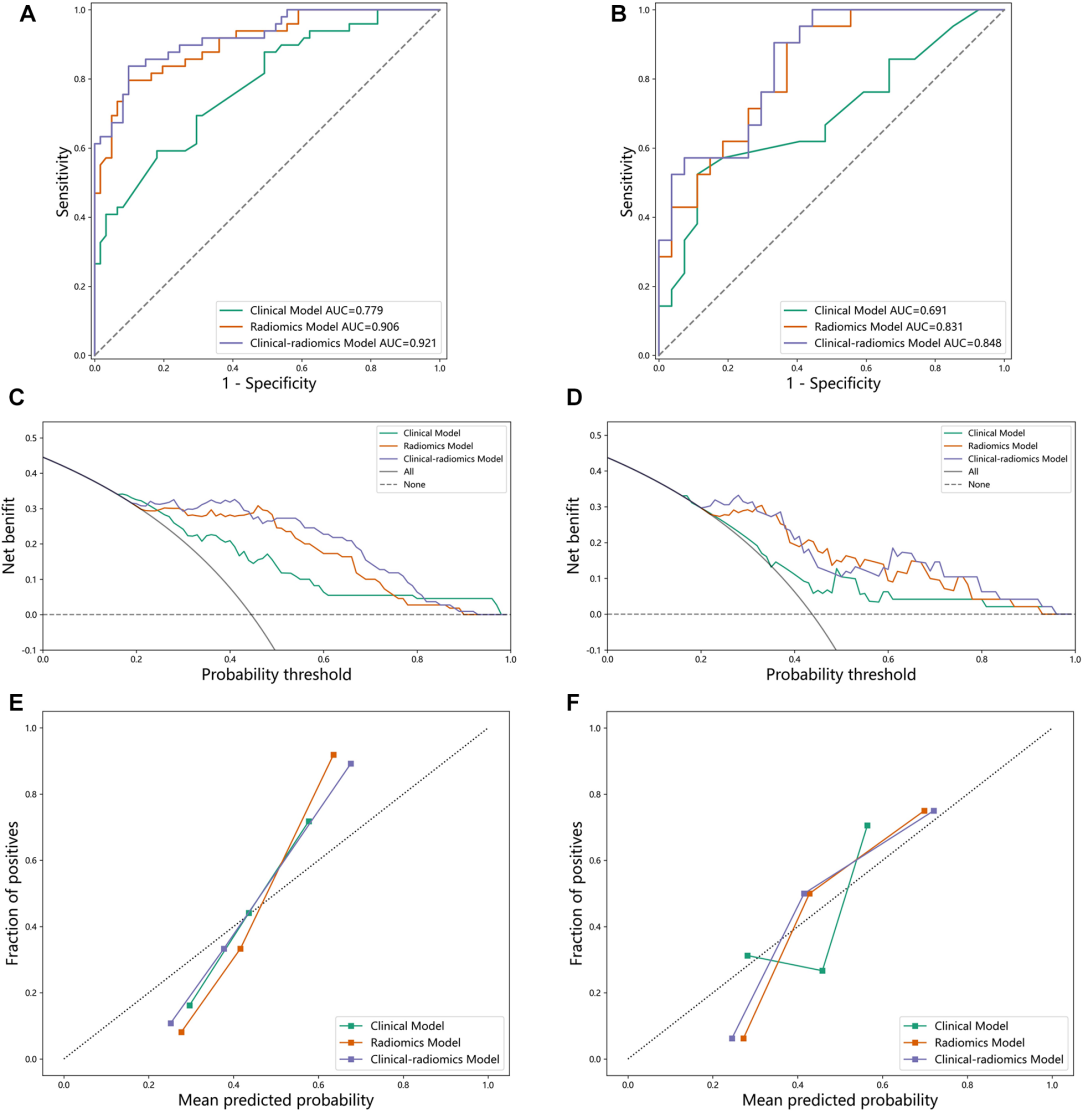
(**A**) ROC curve on the training set. (**B**) ROC curve on the test set. (**C**) decision curve on the training set. (**D**) decision curve on the test set. (**E**) calibration curve on the training set. (**F**) calibration curve on the test set.

After feature screening, the final optimal set of features consists of two parts: three CCTA radiomics features and one clinical feature. When the relative contributions of radiomics features and clinical features to disease judgment were uncertain, we developed three random forest models: a radiomics random forest model based on radiomics features only, clinical random forest model based on clinical features only, and a combined model named HML incorporating both feature types. The AUC, accuracy, sensitivity, specificity of the three models were compared to determine whether the selected features contributed to the diagnosis of coronary heart disease. The training set results are shown on the left and the test set results are shown on the right (Figure 4). We can see that the HML achieved higher AUC in the training set and test set (Figures 4A,B), which were 0.921 and 0.848, respectively. Figures 4C,D are calibration curves, and the 45° diagonal line represents the ideal calibration. The HML is closer to the ideal calibration curve, indicating that the model has better consistency between the average prediction rate and the actual probability. Figures 4E,F are the decision curves, the *X*-axis is the risk threshold, and the *Y*-axis is the net benefit. The black line represents the assumption of no lesion and the gray line represents the assumption of complete lesion. The further away the curve is from both the black and gray lines, the higher the net benefit of the model.

The model that uses only a single clinical feature has the worst performance across all metrics, particularly with a sensitivity of 0.551, indicating its limited ability to detect diseases accurately. Benefiting from the diversity of random forests, the HML that combines clinical and radiomics features consistently outperforms the other two models. Therefore, these comparison results demonstrate the powerful ability of combine random forest in coronary disease classification. By analyzing the comparison results of these three models (Table 4), we can see that:

**Table 4:** Comparison of performance on training and test.

Model		AUC (95%CI)	ACC (95%CI)	SEN (95%CI)	SPE (95%CI)
Train	Clinical	0.756 (0.668–0.845)	0.718 (0.715–0.722)	0.551 (0.412–0.690)	0.852 (0.763–0.941)
	Radiomics	0.906 (0.851–0.961)	0.845 (0.843–0.848)	0.776 (0.659–0.892)	0.902 (0.827–0.976)
	Combine	**0.921** (0.871–0.971)	0.873 (0.871–0.875)	0.837 (0.733–0.940)	0.902 (0.827–0.976)
Test	Clinical	0.619 (0.457–0.781)	0.604 (0.594–0.614)	0.571 (0.360–0.783)	0.630 (0.447–0.812)
	Radiomics	0.831 (0.719–0.943)	0.729 (0.721–0.737)	0.714 (0.521–0.908)	0.741 (0.575–0.906)
	Combine	**0.848** (0.742–0.954)	0.729 (0.721–0.737)	0.762 (0.580–0.944)	0.704(0.531–0.876)

The bold values indicates that the hybrid model proposed in this paper can achieve the state of art effect in the AUC index no matter in the training process or the testing process.

The HML has the highest AUC, sensitivity, specificity of 0.848, 0.762 and 0.704 respectively, which proves the effectiveness of the HML.

Compared with the most advanced multiple logistic regression model, HML easily exceeds the logistic model by 6.5% in AUC, effectively proving that the proposed method can better classify coronary heart disease and provide effective help for doctors.

The effectiveness of the HML indicates that the radiomics characteristics and clinical characteristics of myocardial CCTA can be used as biomarkers for myocardial diagnosis.

## 4 Discussion

The aim of this study is to construct an efficient diagnostic model for coronary heart disease by leveraging radiomics and machine learning techniques to extract features from CCTA images, which are then combined with statistical results derived from clinical features. CCTA is a safe, reliable and non-invasive method. However, the analysis of a large number of CCTA images requires support from medical and human resources. Therefore, it is necessary to combine other methods to process CCTA more effectively. Studies have shown that radiomics can transform images into mineable data information, and then conduct high-throughput quantitative analysis, capturing those difficult-to-detect features in CCTA images.

In addition, we have also integrated clinical data with statistical method to obtain clinical features that exhibit a strong association with the onset of coronary heart disease. Then we utilized machine learning techniques to combine the two sets of features and establish three random forest models with varying feature combinations, ultimately resulting in the optimal feature set. The results of the study showed that the combination of clinical and CCTA features produced the best results, with AUC values of 0.921 and 0.848 for the training set and the test set, respectively. Similarly, Zhao et al. (28) obtained AUC values of 0.914 and 0.827 for the training set and the test set. The higher AUC values may be due to the fact that we combined clinical features and used more data sets, resulting in more extracted features.

In a word, both clinical and CCTA features’ weight should be considered, along with modeling on more diverse data in future study. Therefore, further advancement of our research requires prospective and large-scale randomized controlled clinical studies to better evaluate its clinical application value.

Overall, the effectiveness of our method suggests that radiomics and clinical characteristics of myocardial CCTA can serve as biomarkers for myocardial diagnosis.

## Data Availability

The raw data supporting the conclusions of this article will be made available by the authors, without undue reservation.
